# Glomeruloid haemangioma in Erdheim–Chester disease: An atypical skin manifestation associated with elevated vascular endothelial growth factor‐A levels

**DOI:** 10.1111/bjh.70627

**Published:** 2026-06-19

**Authors:** Jerome Razanamahery, Matthias Papo, Francesco Pegoraro, Felix Blaison, Paul Legendre, Valentin Carado, Martin Caretti, Aubriot Lorton Marie Helene, Jean‐Francois Emile, Augusto Vaglio, Julien Haroche

**Affiliations:** ^1^ Department of Internal Medicine and Clinical Immunology Dijon University Hospital Dijon France; ^2^ Department of Internal Medicine 2, French Reference Center for Histiocytosis Pitie Salpetriere Hospital Paris France; ^3^ Department of Experimental and Clinical Medicine University of Florence Florence Italy; ^4^ Department of Internal Medicine Bordeaux University Hospital Bordeaux France; ^5^ Clinical Immunology Department Le Mans Hospital Le Mans France; ^6^ Department of Dermatology Dijon University Hospital Dijon France; ^7^ Department of Pathology Dijon University Hospital Dijon France; ^8^ Department of Pathology Ambroise Pare Hospital Boulogne‐Billancourt France

**Keywords:** angioma, ECD, histiocytosis, VEGF‐A

## Abstract

Glomeruloid haemangiomas and extensive angiomas occurred in a small subset of Erdheim–Chester disease patients, all showing markedly elevated vascular endothelial growth factor‐A (VEGF‐A) levels despite the absence of POEMS (polyneuropathy, organomegaly, M‐spike, and skin disease) syndrome. Molecular analyses demonstrated mitogen‐activated protein kinase (MAPK)‐activated histiocytes without endothelial mutations, supporting a *paracrine VEGF‐A–driven angiogenesis* mechanism underlying these atypical cutaneous lesions.

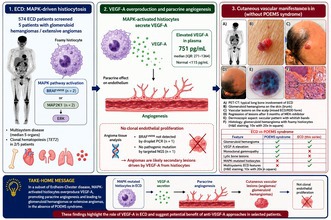

To the Editor,

Erdheim–Chester disease (ECD) is a rare clonal histiocytosis of the ‘L‐group’ characterized by long bone osteosclerosis, peri‐renal infiltrate and/or vascular sheathing.^1^ Additionally, some patients exhibit cutaneous involvement, commonly presenting as eyelid xanthelasma‐like lesions.^2^ ECD is driven by somatic mutation of mitogen‐activated protein (MAP) kinase (MAPK) pathway genes,^3^ particularly the *BRAF* ^*V600E*^,^4^ which promotes proliferation and resistance to apoptosis.^3^ However, other signalling pathways and protein overexpression contribute to the disease manifestations.^3^ One such protein, vascular endothelial growth factor‐A (VEGF‐A), is a pro‐angiogenic factor elevated in both blood samples and tissue biopsies of ECD patients.^5^ Interestingly, VEGF‐A is also elevated in POEMS (polyneuropathy, organomegaly, M‐spike, and skin disease) syndrome, a haematological condition related to monoclonal gammopathy, mostly immunoglobulin M (IgM) with λ light chain,^6^ where patients develop glomeruloid haemangiomas and lytic bone lesions. Legendre et al. previously reported an ECD patient with haemangiomas presenting with elevated VEGF‐A levels.^7^ Herein, we present a series of five ECD patients who presented glomeruloid haemangiomas or extensive angiomas and elevated VEGF‐A levels, in the absence of POEMS syndrome.

We analysed clinical charts from three major referral care centres for ECD in France (Dijon University Hospital and Pitié‐Salpêtrière Hospital) and Italy (Meyer Children Hospital Scientific Institute for Research, Hospitalization and Healthcare, Florence) to analyse the frequency of skin involvement in patients with ECD. Thereafter, we identified patients with haemangiomas or angiomas and no features of POEMS syndrome.

The diagnosis of ECD was based on current guidelines and confirmed histology.^8^ Detection of *BRAF* ^*v600E*^ was performed by picodroplet digital polymerase chain reaction (PCR).^9^ Targeted next‐generation sequencing (NGS) was performed in *BRAF* ^*v600E*^‐negative patients. The targeted molecular analysis was performed on histiocytic infiltrate and bone marrow/blood analysis for clonal haematopoiesis assessment. The targeted NGS screening used the Custom Amplicon Low Input Kit and MiSeq (Illumina), and included *ASXL1, BCOR, BCORL1, CALR, CBL, CSF3R, DNMT3A, ETV6, EZH2, FLT3, GATA2, IDH1, IDH2, JAK2, KIT, KRAS, MPL, NIPBL1, NPM1, NRAS, PHF6, PTPN11, RAD21, RIT1, RUNX1, SETBP1, SF3B1, SMC1A, SMC3, SRSF2, STAG2, TET2, TP53, U2AF1, WT1, ZRSR2*.

For haemangioma, formalin‐fixed paraffin‐embedded sections were stained with hematoxylin and eosin (H&E) and with antibodies against cluster differentiation CD31, CD34 and eryhtroblast transformation specific regulated gene (ERG) to confirm vascular lineage. VE1 immunohistochemistry (IHC) was considered for spatial localization but could not be performed on angioma tissue when histology confirmed the absence of histiocytic infiltration.

The study was approved by the local ethics committee of each participating institution and followed the principles of the Declaration of Helsinki.

We analysed the clinical charts of 574 ECD patients. Among these, 189 patients (32%) had skin involvement. We identified five male patients with glomeruloid haemangiomas or extensive angiomas at the diagnosis of ECD (including the patient reported by Legendre et al.). The patient previously reported by Legendre et al. is included in this series and did not have monoclonal gammopathy; therefore, POEMS syndrome was excluded in all five patients.

The median age at diagnosis was 64 years (interquartile range [IQR]: 47–77). All patients had a multisystemic disease with a median of five affected organs (IQR: 3.5–7). The most commonly involved organs were the bones (5/5), the cardiovascular system (3/5) and the peri‐renal area (3/5).

Regarding the skin lesions, the patients had extensive glomeruloid haemangiomas (*n* = 3) or extensive angiomas (*n* = 2) with various locations. The lesions were mainly located in the trunk (4/5) and were painless and non‐associated with itchiness. One patient had cephalic lesions with extension into the skull. All skin lesions were well‐circumscribed (*n* = 5), reddish (*n* = 5), and some had increased vascularization (*n* = 2). Only one patient had peri‐orbital xanthelasma‐like lesions. Positron emission tomography and computed tomography (PET‐CT) showed typical bone lesions of ECD, and no patients had lytic lesions suggestive of POEMS syndrome. No patient had IgM gammopathy or elevated lambda light chain suggestive of POEMS syndrome. Biological tests showed normal blood counts in all patients, and all had elevated C‐reactive protein levels (median [IQR]: 46 mg/L [10–51]).

Angioma biopsy was performed in three patients and showed glomeruloid haemangioma architecture. In two cases, IHC for CD31, CD34 and ERG confirmed vascular lineage (Figures S1–, S4). To explore a potential clonal relationship between the angiomas and the histiocytosis, we performed molecular analysis on two independent angioma samples. In the first patient (*BRAF* ^*V600E*^ patient), deoxyribonucleic acid extracted from the angioma (histology confirmed the absence of histiocytic infiltration) was negative for *BRAF* ^*V600E*^ by picodroplet digital PCR, while *BRAF* ^*V600E*^ was detected in the patient's histiocytic infiltrate.

In a second patient, targeted NGS was performed on the angioma tissue and revealed no detectable mutations in the targeted panel, including MAPK pathway genes.

Targeted sequencing on histiocytic infiltrate identified *BRAF* ^*v600E*^ for two patients and *MAP2K1* mutation in two patients. Two patients had clonal haematopoiesis with *TET2* mutation detected in blood/bone marrow sample. VEGF‐A was elevated in four patients (median [IQR]: 751 pg/mL [271–1364]; *N* <115 pg/mL) and not available for one patient.

Most patients received targeted therapy (3/5) and others received interferon leading to a metabolic response. Despite the overall metabolic response, the skin lesions were permanent except for the patient with cephalic lesions, which disappeared after mitogen‐activated extracellular kinase (MEK) inhibitor therapy.

The patient's characteristics and images are reported in Table 1 and Figure 1.

**TABLE 1:** Characteristic of ECD patients with glomeruloid haemangiomas or extensive angiomas.

	Age	Sex	Type of histiocytosis	Location of histiocytosis	Mutational status	Type of vascular outgrowth	Location of skin lesion	VEGF (pg/mL) *N* <115	CRP (mg/L)	Monoclonal gammopathy	Kappa light chain and titre (*N* <19 mg)	Lambda light chain (*N* <27 mg)	Treatment	Skin response	Overall response
Patient 1	44	Male	ECD	Bones, mesentery, cardio‐vascular, endocrine, peri‐renal, sinus	No mutation	Nodular and papular: cherry angioma	Trunk	852	46	0	22 mg	0	Interferon	Stable lesions	Stable metabolic disease
Patient 2	69	Male	ECD	Bone, skin (xanthelasma) sinus	*BRAF* ^*V600E*^	Papular: cherry angioma	Trunk	1534	47	0	34 mg	30 mg	BRAF inhibitor	Stable lesions	Partial metabolic disease
Patient 3	50	Male	ECD/RDD	Bone, lymph node, CNS	*MAP2K1*	Nodular: glomeruloid haemangioma	Head, trunk	NA	NA	NA	NA	NA	MEK inhibitor	Response	NA
Patient 4	86	Male	ECD	Cardiovascular, bone, peri‐renal	*BRAF* ^*V600E*^	Nodular: glomeruloid haemangioma	Trunk	650	51	IgG kappa	27 mg	0	BRAF inhibitor		Partial metabolic disease
Patient 5	64	Male	ECD	Bones, peri‐renal, mesentery	*MAP2K1*	Papular: glomeruloid haemangioma	Trunk	145	12	IgG kappa	36	0	MEK inhibitor	Stable lesions	Partial metabolic disease

*Note*: In the location of histiocytosis collum, we have only considered xanthelasma as typical skin lesions. Nodular >1 cm and papular <1 cm.

Abbreviations: CNS, central nervous system; CRP, C‐reactive protein; ECD, Erdheim–Chester disease; GMEK, mitogen‐activated extracellular kinase; IgG; immunoglobulin; NA, not available; RDD, rosai‐dorfman disease; VEGF, vascular endothelial growth factor.

**FIGURE 1:**
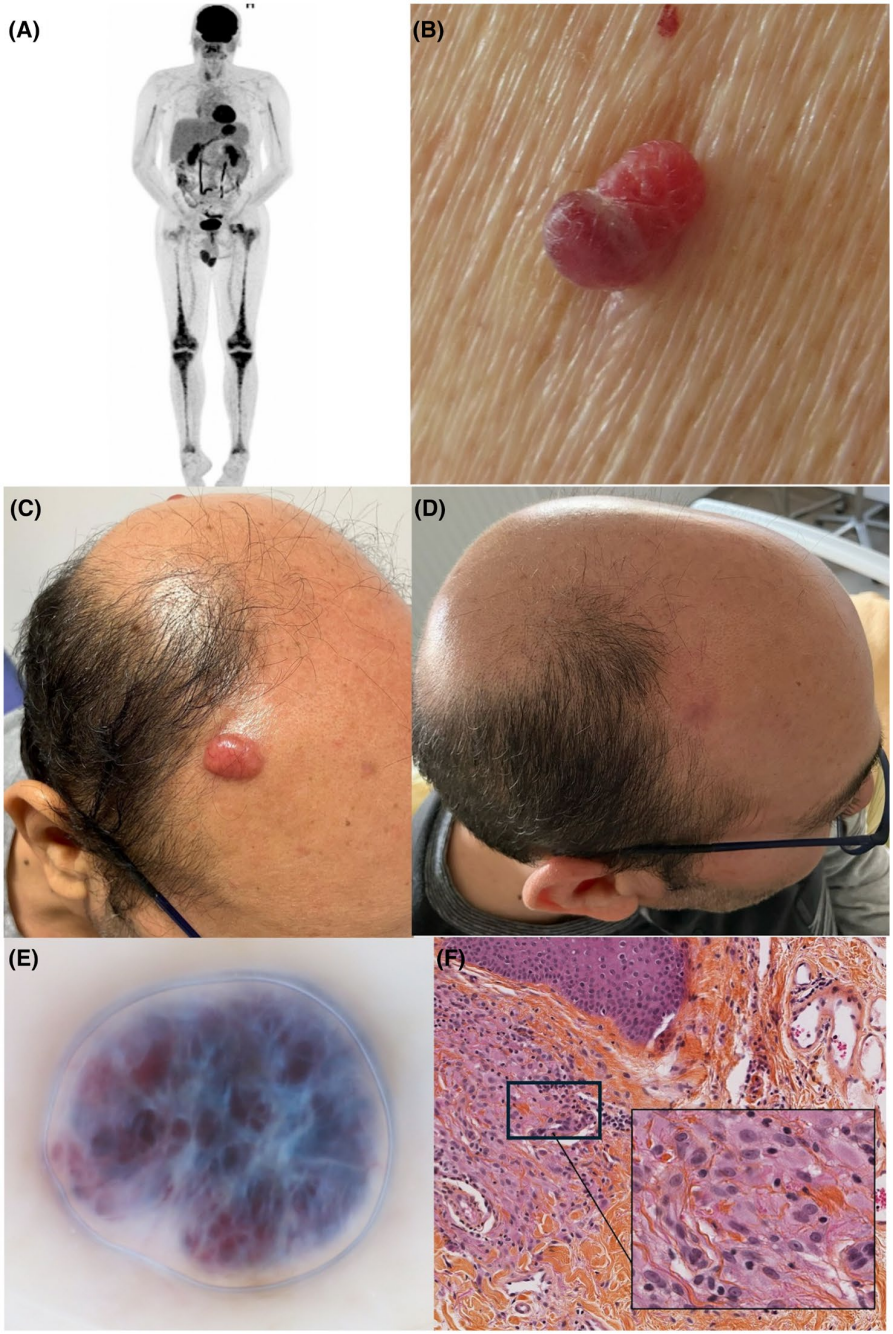
Dermatological presentation of Erdheim–Chester disease (ECD) patients with angioma. (A) PET‐CT showing typical long bone involvement of ECD. (B) Clinical aspect of glomeruloid angioma in a patient with ECD. (C) Vascular related lesions on the scalp in a patient with mixed ECD/RDD form. (D) Regression of the lesions after 3 months of MEK inhibitor. (E) Dermoscopical aspect of glomeruloid angioma (GH), saccular pattern with whitish bands. (F) GH histology with foamy histiocytes colocalization, H&E staining (objective ×10 and objective ×20 in square).

This study presents the first case series of ECD patients exhibiting glomeruloid haemangiomas or extensive angiomas associated with elevated VEGF‐A levels, without POEMS syndrome criteria. The findings emphasize the importance of VEGF‐A in the pathophysiology of ECD, particularly in cases involving atypical skin manifestations. VEGF‐A is a protein encoded by the VEGF gene with a predominant role in angiogenesis and vascular remodelling.^6^ Its increase is reported in POEMS syndrome,^10^ a haematological condition driven by monoclonal gammopathy in which the patient presents skin changes. Roeser et al. reported VEGF‐A elevation in ECD patients, in particular with cardiovascular involvement, suggesting its vascular role irrespective of the underlying conditions. Haemangiomas are characteristic lesions in POEMS syndrome.^11^ They resemble renal glomeruli related to proliferative endothelial cells. They present as vascular papules or nodules, commonly located in the upper body. They can also present as polypoid or scattered vascular lesions.^12^ In our ECD series, the haemangiomas and the angiomas were reddish papules and nodules, located in the upper body, with one cephalic presentation. Interestingly, these lesions were painless and non‐pruritic, distinguishing them from typical inflammatory skin lesions. The shared characteristics with POEMS patients suggest the role of VEGF‐A in the genesis of these lesions in ECD patients. The lack of peri‐orbital involvement in most patients differentiates these cases from the common eyelid xanthelasma. Interestingly, three patients harboured mutations of the MAP‐kinase pathway gene (two *BRAF* ^*V600E*^, one *MAP2K1*), and it is now acknowledged that most ECD patients have constitutional activation of this pathway. The MAP kinase pathway plays a significant role in upregulating VEGF‐A through transcriptional activation and messenger ribonucleic acid (mRNA) stabilization.^13^ These processes are essential for angiogenesis in certain cancers,^14^ particularly in intramuscular capillary‐type haemangioma—a rare infant malignancy frequently associated with mutations in MAP kinase pathway genes.^15^ While MAPK pathway activation can upregulate VEGF‐A in multiple cell types, our data favour paracrine VEGF‐A secretion by MAPK‐activated histiocytes rather than a primary endothelial MAPK‐driven lesion. This conclusion is supported by detection of *BRAF* ^*V600E*^ in histiocytic infiltrates but its absence on the haemangioma tissue by droplet PCR, and the additional absence of detectable mutations on targeted NGS performed on a second angioma sample. Together with the well‐established angiogenic capacity of activated macrophages, these results support a model in which MAPK‐activated histiocytes secrete VEGF‐A, leading to elevated serum VEGF‐A and second local angiogenesis. We acknowledge that VE1 IHC co‐localization (VE1 + macrophage and endothelial markers) would provide additional spatial resolution and recommend this for future studies.

The observed regression of lesions in a patient with both angioma and histiocytic infiltration following MEK inhibitor treatment supports the hypothesis of its upregulatory effect. Nevertheless, the persistence of lesions in some patients receiving targeted therapies suggests that VEGF‐A‐driven angiogenesis may not always respond well to these treatments. This highlights the potential for alternative therapies targeting VEGF‐A, especially for patients with vascular skin manifestations who cannot tolerate kinase inhibitors.

This case series had limitations, mainly due to its retrospective nature and inhomogeneous assessment of patients. Based on the rarity of this manifestation, it was hard to decipher if these lesions were related to ECD or secondary to ageing. Nevertheless, the most compelling argument is the elevation of serum VEGF‐A among these ECD patients with cutaneous manifestations resembling POEMS syndrome in a large ECD cohort.

To conclude, this study describes a novel skin manifestation in ECD patients probably related to VEGF‐A elevation. ECD should be considered by clinicians when multiple new cutaneous vascular lesions appear in the absence of POEMS syndrome. Further biological evaluations are required to better define the nature of these lesions within ECD manifestations and the putative contribution of VEGF‐A in the pathogenesis of histiocytosis.

## AUTHOR CONTRIBUTIONS

**Jerome Razanamahery:** Conceptualization; investigation; writing – original draft; data curation; writing – review and editing. **Jean‐Francois Emile:** Investigation; writing – review and editing; validation. **Paul Legendre:** Investigation; writing – review and editing. **Felix Blaison:** Investigation; writing – review and editing. **Julien Haroche:** Conceptualization; writing – review and editing; validation; visualization; supervision. **Aubriot Lorton Marie Helene:** Validation; formal analysis; writing – review and editing. **Martin Caretti:** Investigation; writing – review and editing. **Francesco Pegoraro:** Investigation; writing – review and editing; validation. **Matthias Papo:** Conceptualization; investigation; validation; visualization; writing – review and editing. **Augusto Vaglio:** Investigation; writing – review and editing. **Valentin Carado:** Investigation; writing – review and editing.

## CONFLICT OF INTEREST STATEMENT

The authors have no conflict of interest related to this work.

## Supporting information


**Figure S1.** Angioma histology in one patient on H&E staining showing vascular lesions.

**Figure S2.** Angioma histology of the same patient showing ERG and CD34 expression in line with vascular proliferation.

**Figure S3.** Angioma histology of another patient on H&E staining showing vascular lesions.

**Figure S4.** Angioma histology of the same patient showing ERG and CD34 expression in line with vascular proliferation.

## Data Availability

The data that support the findings of this study are available from the corresponding author upon reasonable request.
